# Improving the quality of clinical training in maternal and neonatal health in undergraduate nursing students: a participatory action research

**DOI:** 10.1186/s12912-024-02160-z

**Published:** 2024-09-12

**Authors:** Akramsadat Sadat Hoseini, Fatemeh Ghasemi, Fatemeh Valizadeh, Nahid Dehghan Nayeri, Tahereh Toulabi, Shirin Hasanvand

**Affiliations:** 1 Social Determinants of Health Research Center, School of Nursing and Midwifery, Lorestan of Medical Sciences, Khorramabad, Iran; 2 Pediatric Nursing Department, School of Nursing and Midwifery, Tehran University of Medical Sciences, Tehran, Iran; 3 Nursing and Midwifery Care Research Center, Tehran University of Medical Sciences, Tehran, Iran; 4 Social Determinants of Health Research Center, School of Nursing and Midwifery, Lorestan of Medical Sciences, Khorramabad, Iran

**Keywords:** Clinical training, Nursing, Student, Action research, Maternal and neonatal health course

## Abstract

**Background:**

Improving the quality of clinical training is synonymous with accomplishing nursing education goals and improving the quality of nursing care. This study aimed to improve the quality of clinical training in Maternal and Neonatal Health (MNH) in nursing students.

**Methods:**

This action research was conducted in two cycles from June 2017 to June 2018. The study setting was the School of Nursing and Midwifery of Lorestan University of Medical Sciences in southwest Iran. The participants were nursing students, faculty members, clinical trainers, educational managers, and teaching personnel. In the first cycle, semi-structured interviews were held with stakeholders to identify clinical problems and improvement strategies. Based on the results of these sessions, the changes needed to improve the quality of clinical training were planned and implemented. The weaknesses and strengths of the implemented changes were then identified through group discussions with the stakeholders. In the second cycle, a second plan was carried out to correct the weaknesses of the changes planned in the first cycle, and the modifications were implemented and evaluated.

**Results:**

The main three categories extracted included an unsystematic curriculum and inadequate monitoring, inadequate resources and facilities, and the student’s lack of motivation. The measures taken for improvement included holding communication workshops, developing and internalizing logbooks, reducing the number of students in clinical training groups, using modern clinical training methods, and changing clinical evaluation methods.

**Conclusion:**

Improving communication skills among the students, trainers, and hospital personnel and using modern clinical training methods, such as conceptual maps, triple jumps, and clinical skill centers, are the best strategies for improving clinical training in MNH nursing students.

## Background

Maternal and neonatal deaths are key indices showing countries’ health and hygiene status. Improving the level of care is the best way to reduce maternal and neonatal mortalities [1]. As healthcare providers, nurses are the backbone of health systems [2]. Nursing schools aim to train nurses capable of providing clinical services following modern scientific advances and meeting the community’s needs [3].

Clinical training has a significant role in achieving this goal as the heart of nursing education [4–6]. Management is essential since students’ clinical training is complex [7, 8]. In a study based on the opinion of nursing graduates, 80% of the skills acquired during their studies were average for the work environment [9]. Also, evidence suggests that clinical nursing faces many challenges and problems in various domains, including MNH [10–13].

Based on the literature, the teaching quality of the mother and newborn course is in the last ranks of the undergraduate nursing courses, and the average sufficiency of the internship of this course for the acquisition of clinical skills of the students from the perspectives of the teachers in the rank after the fundamentals of nursing, management, community health, psychiatric nursing, pediatric and medical - surgical nursing. In comparison, this lesson has particular importance for the health of society [12, 14]. This is while the neonatal period is crucial for quality care due to the need for multiple adaptations to compromise with extrauterine life and the vulnerability of the newborn [15]. Lack of standard care is associated with long-term complications during infancy, some of which affect the person until the end of life [16]. Also, physiological changes during pregnancy and after in women entering the role of the mother increase their need for care [17, 18]. The best place for training and improving nurses’ knowledge in caring for mothers and newborn are the theoretical and clinical course of MNH for nursing students. Because at birth and a few hours after birth, the first ones who are in contact with the newborn and the mother and have a significant role in the care and education of these vulnerable groups are nurses; they should prepare this before entering the field of work and get an entire and decent career.

As future nurses, today’s nursing students need to gain the required readiness and competence to provide maternal and neonatal care through improved quality clinical training in MNH. The present study was conducted to improve the quality of clinical training in MNH among nursing students using the action research approach, which combines practice and research and replicates what happens in the real world. This approach is systematic research and an intellectual reflection on the change process [19] and has a significant role in improving the quality of nursing education [20].

## Methods

### Design and setting

This action research was conducted from June 2017 to June 2018 in two cycles, each consisting of four stages: identification, planning, implementation, and evaluation.

The study setting was the School of Nursing and Midwifery of Lorestan University of Medical Sciences in southwest Iran. In this setting, nursing students receive four years of training and start working in hospitals after obtaining their bachelor’s degree. MNH is taught to them as a theoretical and clinical subject. The clinical part of the training was held for 5th and 8th -semester students in maternity, maternal and neonatal wards (51 h each) with the full-time presence of a faculty member or a non-faculty instructor in groups of nine to ten students. Due to cultural, belief-related, and legal issues and to respect the patient’s right to receive the services of a same-sex nurse, clinical training on this subject was solely provided to female nursing students, and no male nursing students received any clinical training in the maternity, maternal and neonatal wards [21].

### Intervention

**Diagram 1:**
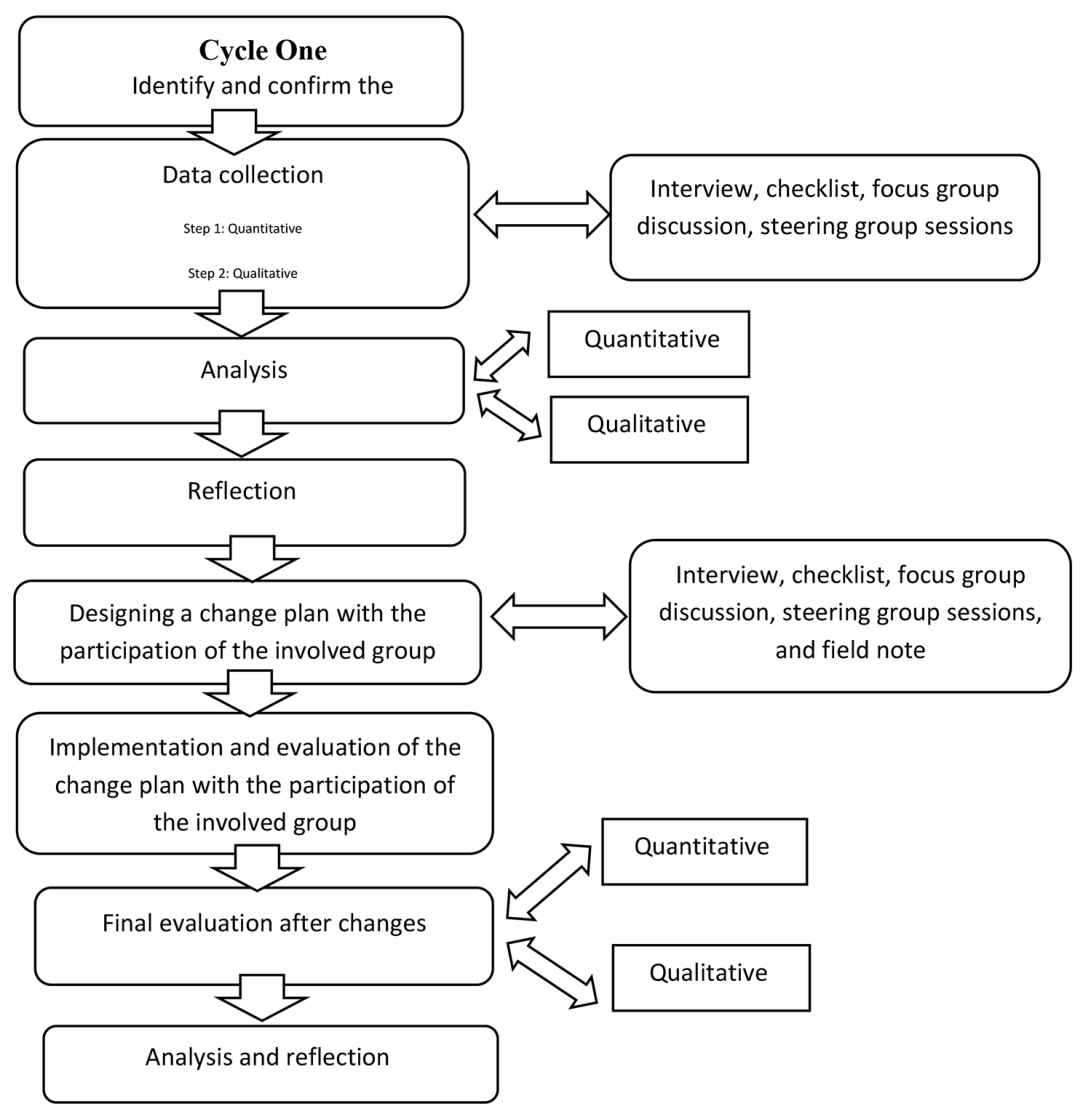
An overview of the steps of participatory action research to improve the quality of MNH courses

The present participatory action research was conducted using quantitative and qualitative approaches in two four-stage cycles, with a reflection on each stage based on Speziale et al. (2011) model. The Speziale model, rooted in participatory action research (PAR) principles, emphasizes collaborative inquiry and reflection and was aligned well with our goal of enhancing clinical training quality through active engagement of stakeholders [17] (Diagram 1).

#### Cycle one

This cycle consisted of identifying the problems and strategies, planning, action for change, and evaluation.

#### Identification

**Step 1** (*quantitative approach*).

This stage aimed to investigate the educational situation of the MNH clinical course. The data was collected with the Iranian questionnaire on the clinical education situation. The whole enumeration method selected students who completed the MNH clinical course (number = 16). The instrument was completed as a self-report. This instrument was divided into two parts: the demographic characteristics of the learners and the second part comprising: 33 items and five dimensions: objectives and curriculum (11 items), teacher performance (9 items), communication with students (4 items), educational environment and resources (5 items) and monitoring and assessment (4 items). Answer questions based on a three-point Likert (yes (3 points), slightly (2 points), and not (1 point)). The results ranged from 33 to 96 to 66 − 33, indicating the unfavorable level, and 99 − 66, the favorable level of clinical education. The mean scores for each domain were calculated by adding the scores for the items and dividing them by the item number. Ghafourifard reviewed and confirmed the validity of the instrument [22]. Computing Cronbach’s alpha confirmed its reliability at 0.99.

**Step 2** (*qualitative approach*).

A qualitative content analysis study was conducted to identify the existing problems of improving the quality of clinical training in MNH. The participants were selected through purposive sampling. They included faculty members, clinical trainers, teaching hospital personnel, and students who had already earned or were earning clinical credits for this subject and had objectively experienced the problems of clinical training. Sampling continued until data saturation was reached. Data were collected through semi-structured, individual, face-to-face interviews and focus group discussions.

The interviews began with the question, “How do you evaluate your clinical training on MNH?” or “Describe your experiences of the clinical training of MNH.” The participants were asked to talk about their problems concerning this subject. The interviews lasted between 45 and 90 min. Focus group discussions were also held to obtain appropriate information, brainstorm, and complement each other’s statements about the problems of MNH training. The groups were homogeneously formed so the participants could freely express themselves in a comfortable setting. The faculty members and personnel formed one group, and the students formed a separate group. One of the research team members took part as the group assistant, and the first author was the facilitator. The group discussion questions were based on the problems stated in the individual interviews and aimed to assess strategies and activities for affecting change. The researcher reminded the participants that the goal of the sessions was not to reach a consensus but to gather data based on their experiences and opinions. Each group consisted of six to eight members. Overall, four 1.5 to 2-hour sessions were held.

All of the interviews were audio-recorded and transcribed verbatim. The data were analyzed in MAXQDA-10 using the approach by Graneheim and Lundman [23]. The problems and strategies were explained and confirmed by the end of stage one. The rigor of qualitative data was confirmed by Lincoln and Guba’s method. The familiarity and professional involvement of the first researcher in the research setting for more than 20 years and the researchers’ long-term engagement and allocation of time for data collection helped gain participants’ trust and thus enriched the data. The research team reviewed, analyzed, and categorized all the codes until the codes, subcategories, and main categories were identified to increase data credibility. The extracted data were then reported to the participants to examine whether they conformed to their experience, and the necessary corrections were applied. Numerous data collection strategies, such as focus groups and semi-structured interviews, helped enrich the data. The external observer method was used to confirm the dependability of the data.

The problems and strategies were explained and confirmed by the end of stage one.

#### Planning

The previous stage’s results were analyzed with two group discussion sessions, and a list of problems was prepared. Then, the challenges were prioritized based on urgency, importance, impact, and ability to solve the problem. Next, strategies and action plans were designed. In this stage, a reform group was formed in the school consisting of the faculty director, the school’s clinical affairs officer, the department head, and faculty members from the pediatrics, maternal and neonatal departments. The first session revolved around funding and supplying the human resources needed to implement the action research for change. The second session was held to decide how clinical training should be carried out based on the plans proposed by the participants.

#### Action for change

Actions planned based on participants’ views and with the change group were implemented (Table 1).

**Table 1:** Program for improving the quality of clinical education of the MNH course

Design of change programs	Participants
Change how people perform an internship.	Department of Pediatrics, Midwifery and students
Preparation of logbook	Department of Pediatrics, Midwifery and lecturer
Reduction in the number of students in Neonatal care internship	Department of Pediatrics, Department of Clinical Education
Conducting healthy newborn internship	Department of Pediatrics, Department of Clinical Education, Officials of the Maternal Maternity Department, and lecturer
Conducting logbook and nursing process workshop	Department of Pediatrics, and Department of Clinical Education
Conducting labor and neonatal resuscitation workshop	Department of Pediatrics, and Department of Clinical Education
Neonatal primary care and familiarization with equipment workshop	Department of Pediatrics, and Department of clinical Education
Providing medical equipment and changing the amount of tuition fees and following up on the notification of the director of the children’s group and new books in the hospital library	The Dean of the Faculty with the Board of Governors of the University

#### Evaluation

Cycle one was evaluated once a week and at the end of the fourth week of the academic semester through individual and group interviews with the stakeholders to reflect on the change implementation process, and clinical training was thereby assessed. The problems and strategies used were evaluated according to the views of the participating trainers and students through two group interviews with the 5th-semester nursing students (four girls) and four professors (three from the pediatrics and one from the midwifery departments), and the weaknesses and strengths of the changes were identified. The comments made by the ward officials and stakeholders were noted and recorded daily, and these groups were invited to participate in the final evaluation interviews if desired. Table 2 summarizes the results related to the planning, implementing, and evaluation strategies proposed for improving the quality of clinical training in MNH.

**Table 2:** The stage of the evaluation of cycle one of action research for improving the quality of clinical training in MNH in undergraduate nursing students

Strategy	Strengths	Weaknesses
Preparation of logbook holding a coordination meeting to familiarize the trainers and students with the concept of logbooks Uploading the logbook on the school website Posting job description and goals on an educational board in the clinical setting Reducing the number of studentsby3-5 Revising the neonatal internship program to familiarize the students with healthy neonates before their familiarization with sick ones Preparing an internship monitoring program active at least 3–4 times during each semester by the department manager and the clinical affairs officer plus the training supervisors Using faculty members as trainers in internships or using trainers with academic and practical experience and knowledge and familiar with the training environment, if in a shortage of faculty members Retaining the trainers Holding workshops for the trainers’ familiarization with job descriptions, training goals, professional ethics and communication Coordination with the trainers to have a close communication with faculty members through a telegram group (With the maternal and neonatal group manager as the group admin)	A specified job description and goals of students and trainer specified expectations from the trainers and students Cooperation between the officials, personnel and physicians in student training Observing sequence and order in the internship program reducing the students’ stress in dealing with a sick neonate Motivation for teaching and learning in the trainers and students, feeling the presence of a supporter Retaining the trainers and increasing the clinical participation of faculty members Empowerment of the trainer for ethical commitment, communication and training goals	Completing the logbook is time-consuming Financial pressure on the school due to the addition of training groups Increased workload of the members of the maternal and neonatal group
Holding a meeting to connect training and clinical practice Holding workshops for the personnel of teaching hospitals on communication, professional ethics and commitment and their job descriptions Rewarding the personnel involved in student training in writing format by the school The department manager receiving feedback from those in charge and the head nurses of the ward during the internship program Reducing the number of students in clinical settings Full-time supervision of the internship by the trainer	Motivating the personnel Giving retraining and in-service training points for participation in training workshops Generating commitment in the officers and head nurses	The reluctance of a few personnel to attend the workshops
Preparing a logbook Inclusion of the students’ training activities in the evaluation	Dividing the evaluation into predetermined items and specifying the evaluation criteria in the logbook for the students and trainer Inclusion of the students’ activities, including group discussions	
Preparing suitable training environment and resources Interaction of the faculty members in requesting newly-published books based on their needs through coordination with the training supervisor Hospital managers providing training equipment Dedicated break from work for the students in the hospital restaurant, the use of the training space and equipment available in the school’s clinical skills laboratory for neonatal examination and resuscitation training and familiarization with the stages of childbirth before entering the clinic	Updating library books decrease students’ stress Increased patient safety Limited student welfare facilities	
Holding training workshops for ethics, commitment and communication geared toward the students Recruiting competent, interested and motivated trainers	Proper interaction of the medical and training teams	

#### Cycle two

The second cycle was carried out in four stages to correct the changes implemented in cycle one. This cycle also included identification (the weaknesses and strengths of cycle one), planning, action to repair, and evaluation stages. The second cycle started from the eighth week with two change program working groups consisting of MNH group managers and representatives of students and professors who had experienced the change program to strengthen the strengths and improve the weaknesses of the strategies implemented in the first cycle. Two group interviews were done with students, instructors, and staff in this phase.

Following the evaluation of cycle one and determining the strengths and weaknesses of the implemented changes, two focus groups of stakeholders were formed to provide corrective recommendations. Changes were then planned and implemented based on these recommendations.

### Ethical considerations

This AR was approved by the Research Ethics Committee of Lorestan and Tehran Universities of Medical Sciences. All the participants were informed of the study’s objectives and were free to express or withhold information. The duration of the interviews was set based on their physical and psychological states. They were reminded that they could end the interview if they felt uncomfortable or unwilling to continue. The interviews were conducted in an appropriate and peaceful setting within a teaching hospital or school of nursing with respect for the subjects’ privacy. After obtaining the participants’ permission, the interviews and sessions were recorded. All the audio and video recordings from the focus group discussions and interviews were only accessible to the researchers.

### Rigor

The familiarity and professional involvement of the first researcher in the research setting for more than 20 years and the researchers’ long-term engagement and allocation of time for data collection helped gain participants’ trust and thus enriched the data. The research team reviewed, analyzed, and categorized all the codes until the codes, sub-categories, and main categories were identified to increase data credibility. The extracted data were then reported to the participants to examine whether they conformed to their experience, and the necessary corrections were applied. Numerous data collection strategies, such as discussions in focus groups and semi-structured interviews, helped enrich the data. The external observer method was used to confirm the dependability of the data. Two nursing professors outside the research team who were experts in qualitative research were given excerpts from the interview and group discussion transcripts and examples of the process of extraction of the preliminary codes, categories, and sub-categories to examine and give their feedback; their suggestions were applied, and the procedure was thus confirmed.

## Results

### The first cycle

Tables 3 and 4 present the demographic of the participants. Table 5 shows the frequency distribution of students’ views on the clinical education state of the MNH course before the change.

**Table 3:** Demographic characteristics of the participants before and after the change (quantitative identification stage)

Variables	Description	Before		After	
		*N*	(%)	*N*	(%)
Age (year)	21	1	6.3	0	0
	22	11	68.7	0	0
	23	3	18.7	16	76.2
	24	0	0	4	13.8
	25	1	6.3	0	0
Gender	Female	16	100	20	100
	male	0	0	0	0
Marital status	Single	14	87.4	17	85
	Married	2	12.6	3	15
Term	8	16	100	20	100

**Table 4:** Demographic characteristics of the participants before and after the change (qualitative stage of identification)

Variables	Description	Before		After	
		*N*	(%)	*N*	(%)
Age	24 − 20	7	10	7	10
	30 − 25	4	7/5	4	7/5
	35 − 31	8	4/11	8	4/11
	41 − 36	5	1/7	5	1/7
	42≥	6	6/8	6	6/8
Gender	Female	30	100	30	100
	male	0	0	0	0
Instructor	Faculty	5	7/16	5	7/16
	Non -faculty	6	20	6	20
Personnel	Matron	2	7/6	2	7/6
	Supervisor	4	3/13	4	3/13
	Head nurse	2	7/16	2	7/16
Degree	Bachelor	22	4/31	22	4/31
	Master	5	1/7	5	1/7
	PhD	3	9/4	3	9/4
Marital status	Single	14	7/46	10	3/14
	Married	16	3/53	15	4/21

**Table 5:** Comparing the frequency distribution of students’ views on the clinical situation of the MNH course before and after the change

Quality Dimensions	Before		After	
	Favorable Frequency (%)	Unfavorable Frequency (%)	Favorable Frequency (%)	Unfavorable Frequency (%)
Objectives and curriculum	13(81.2)	3(18.8)	(85)17	(15)3
Teacher performance	13(81.2)	3(18.8)	(100)20	-
Communication with students	10(62.5)	6(37.5)	(100) 20	-
Educational environment and resources	2(12.5)	14(8.5)	(80) 16	(20) 4
Monitoring and assessment	3(18.8)	13(81.2)	(100) 20	-
Total	3(18.8)	13(81.2)	(100) 20	-

A total of 680 initial codes were obtained from the analysis of the thirty interviews. Reviewing the initial codes and categories led to extracting three main categories. Table 6 presents the main categories and subcategories.

**Table 6:** The categories and subcategories extracted from the study for the identification phase

Category	Subcategory	Code
Unsystematic curriculum and its monitoring	Unsuitable curriculum Unstructured and inappropriate evaluation Trainer inefficiency	Unclear educational goals No monitoring of clinical education Lack of sequence and order in the internship program The students not being ready to enter clinical practice Not having an internship program for both genders No male students in the maternal and neonatal ward Training needs and interest in this subject among the male students The male students needing to be present in this practice for their future professional requirements Nurses needing to receive comprehensive maternal and neonatal training Not holding a clinical unit on healthy neonates Both genders needing to learn this topic Student overload in the ward No scoring for the students’ activities Inconsistency between evaluation methods and goals No knowledge about evaluation methods The evaluation method not being transparent The evaluation method not being creative An unfair evaluation An incomplete evaluation A preference-based evaluation – The trainers not differentiating between the students Not taking into account the students’ creative and critical thinking in the evaluations The evaluation being stressful for the students The students not knowing about the evaluation items upon their entry into the ward Trainers ‘inadequate practical/academic experience Trainers ‘inadequate support for the students
Inadequate resources and facilities	Unsuitable training environment Inadequate equipment Inadequate welfare facilities	Conducting group discussions in the personnel’s break room The lack of computer facilities The lack of resources for studying The lack of new and up-to-date books in the library The lack of a space for the students to take a break
Students’ lack of motivation	The lack of interest in the discipline Inadequate competence A tarnished identity Personnel’s beliefs about the nursing profession and nursing students Inappropriate personal and intragroup relationships	The lack of trust in the students The lack of support for the students in times of crisis The students’ humiliation in public Discrimination between the nursing and the medical students Poor communication with the students Feeling like a useless extra person in the ward The use of humiliating words in interaction with the students Choosing the nursing profession for its better career prospects The students’ unwilling to work in the neonatal ward in the future Nursing students as second-class citizens The students’ presence not permitted during specialized procedures and doctor’s visits The students performing routine tasks The non-participation of the students in specialized nursing care

#### Unsystematic curriculum and its inadequate monitoring

This category included the following subcategories: Inappropriate curriculum, unstructured evaluation, and trainer inefficiency. An 8th-semester student discussed the inappropriate curriculum due to the big gap between theoretical lectures and clinical training:

“*We studied the theoretical topics three or four semesters ago and could not remember when we finally went to the ward. It would have been better if we had been reminded of some lectures before entering such sensitive wards as the neonatal and maternity ward*” (N: 20).

The ward head nurse said about the students’ lack of mental and psychological readiness:

“*As soon as they enter the ward and encounter an abnormal infant*,* they get frightened*” (N: 28).

Regarding the lack of information about the training objectives, an 8th-semester student argued:

“*It is not clear what we are supposed to be doing in the ward. Every day is the same and ends the same way. Our goals are unclear during the internship*” (N: 19).

About a large number of students in the internship groups, a trainer argued:

“*We would take a large number of students to the ward*,* and that did not have a good effect on training*,* such that I was unable to provide training to all the students during the day*,* and sometimes the students were even missing from my sight*,* and that means the waste of the day for the students*” (N:30).

Regarding the lack of monitoring by the school, a student explained:

“*During the time we were in the maternity ward*,* we were not monitored*,* neither by the school nor by the hospital*,* and so we could not discuss our problems*” (N: 16).

A student discussed the lack of transparency in the evaluations and argued:

“*It is not clear at all which item we were assessed on. For instance*,* we were all the same at work*,* but the scores were widely different*,* or vice versa. The trainer gives scores in the same range and makes no distinctions between the active and the passive or the disciplined and the undisciplined students*” (N: 4).

Regarding some of the trainers’ outdated information, one student said:

“*The trainers have got to have clinical experience and be scientifically up-to-date for the students to enjoy their internship and be encouraged to learn. I have not experienced this in my internship*” (N: 14).

#### Inadequate resources and facilities

This category comprised inadequate equipment, inappropriate educational space, and inadequate welfare facilities. About the lack of access to educational facilities in hospitals and maternal and neonatal wards, a trainer said:

“*There is no library or a place with computer and internet access*,* or the few that do exist are often disconnected from the internet or have problems*” (N: 3).

Regarding the inappropriate educational space and classrooms for the nursing students, a student stated:

“*We had no classrooms for the group discussions and had to stand in the middle of the ward to give our presentations or in the treatment room*,* where the discussions were frequently disrupted by the personnel coming and going*,* and this disrupted the personnel’s work and insulted the trainer and us*” (N: 19).

A student discussed the lack of proper welfare facilities for the students and said:

“*We have to wander around during our break. A break is meant to relieve our fatigue*,* but it only makes us more tired*,* and then we return. There are also the insults from some personnel*,* who complain that the students are wandering around the hospital*” (N: 19).

#### The student’s lack of motivation

This category included the following subcategories: Insufficient interest in the discipline, inadequate competency, humiliating experiences, tarnished identity, inappropriate personal and intra-group relationships, and the personnel’s beliefs about the nursing profession and nursing students.

Regarding the attitude to working with mothers and neonates, a student explained:

“*I have no interest in working with a parturient or a thumb-sized baby and do not think I will ever be working in these wards later on*” (N: 18).

A student revealed a lack of motivation for learning:

“*I have no interest in nursing*,* and my discipline used to be the computer*,* and I merely turned to nurse because of career prospects*” (N: 4).

Concerning having missed opportunities for learning as a result of the personnel’s actions, a student commented: “*In teaching hospitals*,* the personnel does the work themselves very quickly*,* and the whole work is already done by the time we decide to carry out patient care along with our trainer*,* and we miss even just observing the procedure*” (N: 1).

About the medical team’s lack of cooperation in training the students, one of the trainers complained:

“*Sometimes the neonatal or maternity personnel and physician say that nursing students should not enter the ward for infection control*,* while the case is that we sometimes observe [hygiene rules] better than they do*” (N: 11).

As for the students’ and trainers’ poor professional rapport with the clinical personnel, a hospital nursing director explained:

“*Sometimes*,* the students do not have any respect for ethical and professional issues. For instance*,* they do not introduce themselves or exchange greetings with the personnel. They are in the ward for a few hours without the head knowing what they are planning to do*” (N: 7).

The training supervisor discussed the personnel’s interferences in the trainers’ and students’ work and said: “*Some ward heads do not allow the students or trainers to perform certain care measures*,* such as inserting an IV LINE*” (N: 15).

A student described a nurse’s hostile attitude and improper behavior toward her. She said: “*The personnel did not treat us properly in the maternity ward*,* as if we were unwanted extras. They constantly threw words at us and nagged us*,* ‘do not stand here*,* ‘do not do this*,* ‘do not touch that*,* ‘stand aside*,* ‘Do not block the way*!” (N: 1).

Regarding the discrimination made between the medical and nursing students, a student said:

“*They do not allow me to sit in the nursing station*,* but the medical students easily use the station to review the records and take a break*” (N: 19).

A graduate midwifery trainer stated that the students consider some care procedures trivial:

“*Nursing students feel like taking VS is a low-level task*,* and they feel disrespected when you ask them to do it*” (N: 17).

One of the nurses also discussed how the students trivialize specific care tasks:

“*Most students are disinterested in primary maternal and neonatal care and prefer to perform invasive or medical procedures*” (N: 26).

Following the evaluation of cycle one, the strengths and weaknesses of the implemented changes were determined (Table 2).

### Cycle two

Frequency distribution of students’ views on the clinical education state of the MNH course after the change presents in Table 5. In cycle 2, changes were planned and implemented based on recommendations (Table 7).

**Table 7:** Cycle two of the action research for improving the quality of clinical training in MNH in undergraduate nursing students

Identification and Implementation		Evaluation	
Wasting the trainers’ and students time regarding to the large size of the logbook	Summarizing the logbook and integrating the maternal, maternity and neonatal logbooks into one instead of three	**Strength**	**Weakness**
Failure to internship with sufficient preparation due to fatigue and compacted internships	Use of the triple jump evaluation method The use of concept maps from the admitted patients for preparing a nursing care program	Clarity of training goals and job descriptions, and getting the same treatment by the trainers The better and more long-lasting and attractive nature of the learning method for the students The avoidance of the waste of time and the optimal use of the internship hours for preparing scientific materials Promoted creativity and thoughtfulness in planning care	Some trainers’ negative attitude toward the logbook Some of the patients’ relatives and personnel misunderstanding the use of cellphones for preparing scientific materials The time-consuming and difficult nature of modern methods

The final evaluation results after implementing changes on two occasions based on the focus group interviews showed that the unsystematic curriculum changed to a relatively systematic and satisfactory one, the inefficient trainer became more effective, the poor interactions became reasonably solid, and the poor evaluation became efficient. The student’s lack of motivation was replaced with motivation and interest in working in maternal and neonatal wards.

Two of the students participating in the focus groups commented on the systematic curriculum:

“*Just the fact that a logbook was prepared and we*,* the trainer and the personnel*,* learned our job description at the beginning of the internship*,* that the trainers’*,* personnel’s*,* and student’s expectations from each other became clear*,* these truly abated my stress about doing something that violates the rules and regulations and being penalized for it*” (F2 and N5,1).

One of the participating trainers also discussed the somewhat appropriate interactions formed as a result of the reduced size of the educational groups:

“*The number of students was decreased*,* which enabled me to communicate more effectively with the students and the personnel. I had more opportunities for teaching too. Due to the reduced number of students*,* the physicians’ and personnel’s cooperation also grew*,* and there was no longer any nagging by the physicians and nurses. They even allowed the students to be present during the physicians’ visits and medical procedures”* (F1 and N3).

One participant commented:

“*The implementation of this method was perfect because it allowed us to observe the case and at the same time carry a search on our cell phone*,* because it [i.e.*,* the material] stuck better in our memory and we were no longer forced to study it at night with all that fatigue just to fulfill a duty”* (F2 and N4).

One of the students commented on motivation and interest and said:

“*When the physician allowed us to be present in the visits and tried to engage us and asked questions about the infant and his status and asked what procedures had to be carried out for the infant*,* I felt proud of myself*,* as if I was part of the medical team and contributed to the care given*,* and this made me motivated and interested*,* and so I tried to study and be prepared for responding*” (F1 and N6).

## Discussion

According to the present study, the problems of clinical training in MNH include the unsystematic curriculum and its inadequate monitoring, inadequate resources and facilities, and the student’s lack of motivation (Table 6). The absence of a systematic curriculum and monitoring is a problem in clinical training for nursing students, as confirmed by evidence essentially [6, 24–26]. Stakeholders developed a specific internship logbook per the participants’ suggestions of this action research to create a systematic curriculum, specify the goals, and offer the students and trainers a job description. The transparency of the students’ job descriptions affects their learning and contributes to proper communication with the personnel. In line with the present study, Schüttpelz (2016) argued that a logbook is a valuable tool to make training goals transparent, facilitate trainer-student rapport, and standardize clinical training [27]. Another problem discussed in this category was the inappropriate number of students, given the sensitive and vulnerable nature of neonatal and maternity wards as clinical settings. The participants argued that this factor could increase the risk of infection, make the curriculum organization difficult for the trainer, and create tensions between the students, trainers, and medical personnel. Studies have shown that the main barrier to clinical training is the inappropriate number of students in the ward [26, 28]. Previous studies have shown that a large training group in clinical settings means reduced feedback and can result in negligence of errors and unethical behaviors and disrupt the quality of training and the student’s success [28, 29]. A proposed strategy was to reduce the number of students in the training groups to three to five, which appears more satisfactory for the ward managers, personnel, trainers, and students. This strategy enabled proper interactions between these groups in an indirect form. As the ward became less crowded, stresses abated, and the trainer had more time to spend with the students. Also, the physicians allowed the students to be present in the ward during the medical procedures and visits and involved them in their discussions. Based on the students’ statements, such a strong presence in the clinical setting and no longer being ignored contributed to their self-esteem.

The students were not ready to enter the maternity ward; simulation training was applied to educate them on the stages of childbirth before entering the ward. Jurana et al. (2015) used simulation as an effective active technique for gathering real experience in a controlled and safe environment for students and patients [30]. This technique has high validity in nursing education [31] and improves learning, skill acquisition, and dealing with clinical problems such as student overload in clinical settings [30, 32].

The inappropriate evaluation system was another challenge in clinical training. In line with this study, Norouzi et al. (2016) also reported improper evaluations by undergraduate trainers and their inadequate knowledge about the assessment domains and the expectations that surpassed the training given as some of the students’ experiences in this category. In their study, the students believed that there were no specific criteria for clinical evaluation and argued that they were always evaluated based on the trainers’ personal preferences [24]. According to some evidence, the students’ significant problem in clinical settings included the trainers’ evaluation mode and their lack of sympathy and responsiveness [13, 33]. Thus evident that the clinical evaluation of students requires a more transparent instrument [34, 35]. Based on participants’ suggestions, an evaluation form was placed in the logbook that complied with the students’ job description and training objectives, and the trainers were educated on how to use it.

Trainer inefficiency was another subcategory extracted due to the trainers’ inadequate skills in training (practical and academic), management, communication, student support, and accountability. Studies have shown that insufficient knowledge and experience and the lack of accountability in clinical trainers are the main problems of clinical training for nursing students [6, 25, 26]. Effective clinical trainers make the clinical setting attractive for the students and familiarize them with the spirit of philanthropy, communication skills, and clinical competency [26]. They serve as role-model for them and boost their self-esteem [36]. Similarly, de Swardt et al. argued that clinical trainers could contribute to the students’ tendency toward the nursing profession [37]. According to the participants of this study, the reasons for trainers’ inefficiency were their constant replacement with others in the clinical setting and the late payment of their wages (if not on a fixed salary system). They argued that these factors could lead to the recruitment of inexperienced trainers with horrible work experience.

This study solved this problem by retaining motivated and experienced trainers with adequate knowledge for teaching MNH. Also, a group was formed in one of the web messaging services to promote coordination between the faculty members and clinical trainers. Another solution was to facilitate the timely payment of wages to attract more experienced clinical trainers.

Inadequate resources and facilities were another extracted category. Evidence suggests this problem negatively affects nursing education in different societies [10, 25, 26, 38]. The lack of equipment is considered the cause of nurses’ disappointment in decision-making, delayed care, and emotional tensions. Adequate medical equipment, resources, welfare, and physical facilities are essential in any clinical setting to acquire good professional skills [39]. In the present study, the teaching hospitals’ library books were updated with the cooperation of the hospital directors.

Moreover, through the participation of the training authorities of the hospitals, some classes were dedicated to group discussions between the students and trainers. They were also given any medical equipment they desired, whether for the ward’s needs or not. These accomplishments show that clinical training settings already have these facilities and can provide them to those seeking them. The key to taking advantage of these resources is to request them. The present study facilitated these requests and encouraged the group’s participation in the distribution of resources.

The student’s lack of motivation was another extracted category. The lack of motivation among students at the MNH unit was caused by their lack of interest and desire to learn, lack of clinical qualification, damaged identity, and traditional beliefs about the nursing profession and students. According to Valizadeh et al., students choose nursing more than other fields due to job opportunities, which can result in a lack of focus on the career goals [40]. Providing a sense of belonging is a crucial mission of higher education institutions, especially in the sensitive and vulnerable parts of MNH. This necessitates a committed and skilled instructor based on evidence, as well as the hiring of experienced instructors and a suitable educational atmosphere [41]. The student’s feeling of acceptance, being valuable, and belonging are all impacted by the positive communication between the nurse and the student [42, 43]. Students who lack a positive professional identity will suffer from lower self-confidence, weakened clinical reasoning skills, and difficulties in establishing interpersonal communication. Their sense of belonging to the nursing profession decreases, their motivation to learn decreases, and they will be less flexible and efficient in dealing with care challenges. Evidence suggests that enabling the students to find their role model can help eliminate their disappointment, disinterestedness, and lack of motivation [41, 44]. As a result, this study used fixed trainers with adequate skills to reinforce professional values and make the clinical experience more attractive for the students.

The inappropriate cooperation of clinical nurses and medical teams was another reason for the student’s lack of motivation. One of the objectives of undergraduate nursing curricula is to prepare the students and increase their self-esteem to provide quality healthcare services and cooperate with the care team [46–48]. Cooperation between healthcare providers and universities is essential for preparing the students to assume their nursing role [49] smoothly, engage in socialization and adopt professionalism [48, 50]. The lack of mutual understanding removes the possibility of close and empathetic relationships and turns the students’ period of presence in clinical settings into a bitter and futile experience [45]. In this study, the strategy used to solve this problem and create a suitable clinical environment was to familiarize clinical nurses with their job description in training hospitals by holding educational workshops. Also, holding a coordination meeting to familiarize the trainers and students with the concept of logbooks, uploading it on website, posting job description and goals on an educational board in the clinical setting were considered important (Table 2). Another solution was for the school or hospital to devise an annual incentive scheme for that clinical and medical personnel who effectively engaged in student training.

Another challenge that deprived the nursing students of motivation was inappropriate communication and poor treatment on the part of the training and medical teams, which included an array of humiliating experiences and a tarnished identity. In the learning process, nursing students often face problems in their professional identity [37]. Studies have shown that the student’s energy is partly consumed by the challenges and conflicts of dealing with the clinical personnel and believed that the medical personnel does not accept the students as a group member [6, 26, 45]. The students’ positive and negative experiences and attitudes toward clinical practice depend on the relationship between the training and medical teams [46, 50, 51]. Negative communication and behavioral and verbal aggressions projected onto the students from the trainers and nurses can cause stress and a sense of inefficiency and incompetence in the students and adversely affect the formation and development of their professional identity [52]. Students with a tarnished professional identity will have lower self-esteem and experience problems in their interpersonal relationships, sense of belonging to the nursing profession, and clinical reasoning skills. Inevitably, their motivation for learning will be diminished, and they will be less flexible and efficient when faced with care challenges [53]. The strategy to solve this problem was to hold educational classes for the personnel, trainers, and students on communication, professional commitment, and ethics. Also, frequent meetings were held between the maternal and neonatal group, the head nurses, and training supervisors to discuss training problems, provide solutions, and create harmony between training and the treatment goals.

In the second cycle, the students assisted in promoting the rigor and fairness of the evaluation (Table 7). For this purpose, the students completed an evaluation form for themselves and their classmates. The mean of the student’s self-score, her classmates’ given score, and the trainer’s score were calculated as the student’s final score. Students should have an active role in their evaluation, and if this involvement is achieved correctly, they can better understand their expectations and identify their weaknesses and strengths [26]. The participants also noted the need for creative and critical thinking and clinical decision-making in these evaluations. Nursing education should frequently improve students’ critical thinking, problem-solving, and decision-making skills [54]. Studies have shown that modern evaluation methods significantly generate creative thinking, meaningful learning, and clinical decision-making [55–58]. Conceptual maps and triple jump assessment were used based on participants’ suggestions; through these tools, the students observe a case, research it on their cell phone or in library resources, and present it in class form. Since evaluation was used as a tool for learning creative thinking, this action resulted in more excellent trainer and student satisfaction from the evaluation. The evidence shows that using conceptual maps in nursing education helps improve education [59, 60]. Also, a critical and accessible element for making decisions about patient care is the transparent and judicious use of evidence, which Triple Jumps provides [61].

A strength of this study was the participation of all the stakeholders, including executive managers and officers of treatment and training, professors, trainers, students, head nurses, and training supervisors, which resulted in a deeper understanding of the existing problems and the offering of more practical and accessible solutions with minimum financial costs.

The study limitations included the absence of male students among the participants because male nursing students need to pass clinical training in MNH and instead take clinical training in emergency nursing services. To strengthen the male students’ skills in providing emergency maternal and neonatal care, simulation was used instead of clinical training, and topics including natural childbirth, neonatal resuscitation and examination, and neonatal ward equipment were trained in practice to male students in the clinical skills laboratory.

## Conclusion

The quality of clinical training in MNH among nursing students was improved so that the trainer’s performance in dealing with the student improved. The monitoring and evaluation by education managers and healthcare workers have reached favorable conditions. According to the results, in MNH clinical training, communication skills should be improved, and periodical workshops should be held for the personnel to cooperate with the training staff. Reducing the number of students in educational groups is a key way to enhance education quality, regardless of the financial cost. Also, ongoing interactions among the training and treatment authorities, the participation of competent nursing personnel, and providing a supportive clinical environment for training can help improve the students’ professional and clinical skills. Improving training can also be accomplished by ensuring that the education authorities and professors continually engage in action research activities. Continuous efforts towards quality education should not be hindered by economic problems. If managers are convinced that the quality of nursing services and the role of this group in prevention and health education are important, the mindset that clinical education is the most expensive part of education will be replaced with an investment in the health society. In case of lack of financial ability, training by simulation method, use of new learning and evaluation methods, and use of trainers with high communication skills and aware of the supporting role and counseling and adherence to ethical principles and professional commitment will improve the clinical education of the MNH course. To improve the quality of education, it is important to revise the educational curriculum and increase the unit of MNH in a coherent way, at least in the form of two units, while also paying attention to cultural and gender issues as solutions.

## Data Availability

The datasets generated and analyzed during the current study are not publicly available due to confidentiality but are available from the corresponding author at reasonable request.

## Abbreviations

MNH: Maternal and Neonatal Health
